# Biochar: An effective measure to strengthen phosphorus solubilizing microorganisms for remediation of heavy metal pollution in soil

**DOI:** 10.3389/fbioe.2023.1127166

**Published:** 2023-03-02

**Authors:** Haoming Chen, Hanfeng Jiang, Muhanmaitijiang Nazhafati, Lingli Li, Jinyan Jiang

**Affiliations:** School of Environmental and Biological Engineering, Nanjing University of Science and Technology, Nanjing, China

**Keywords:** phosphate solubilizing microorganism, biochar, heavy metal remediation, soil, protection

## 1 Introduction: Phosphorus solubilizing microbial remediation is a sustainable technology for soil heavy metal remediation

### 1.1 Microbial remediation is an effective measure to achieve sustainable remediation of heavy metals in soil

Heavy metals (HMs) pollution of soil caused by human activities is a serious threat to human health and sustainable social development. The insidious, lagging, long-term, inhomogeneous and irreversible nature of heavy metal pollution has led to serious degradation of soil ecological structure and function. Most importantly, heavy metals in soil can be enriched into animals or humans through the food chain (food crops), threatening human health and life (Jan et al., 2015). Therefore, remediation of heavy metals for soils has been one of the key issues in the field of environmental remediation. Compared with physical or chemical remediation technologies, microbial remediation technology is gradually recognized for its green, low cost, easy operation and long sustainability (Maity et al., 2019). Although most HMs are difficult to degrade and remove by microorganisms in soil, microorganisms (microbial uptake, transformation, mineralization or immobilization) can convert HMs to less toxic forms or reduce their mobility (Kotrba and Ruml, 2000; Lin et al., 2023). Among them, biomineralization is a common and effective method for remediation of HMs pollution in soil, which is mainly through the interaction between microorganisms and HMs to form mineral crystals (such as, phosphate, carbonate, sulfate, arsenate, fluoride, oxide, hydroxide, and manganese oxide, etc.) outside, between or within the cells of microorganisms (Tayang and Songachan, 2021; Lin et al., 2023). Not only that, among up to 60 kinds of biomineralization products, metal phosphates have been of concern due to their high stability.

### 1.2 Mineralization allows phosphorus solubilizing microorganisms (PSM) to be successfully used for the solidification/stabilization of HMs in soil

PSM are known for their ability to convert the insoluble state of phosphorus (e.g., iron phosphorus, calcium phosphorus, highly stable organic phosphorus, etc.) in the environment into a form that can be used directly by organism (Qian et al., 2019; Deb et al., 2016). The recognized mechanisms of phosphorus dissolution by PSM can be classified into two categories according to the type of phosphorus source. Firstly, dissolution of inorganic phosphorus sources by means of low molecular weight organic acid release, proton secretion to generate H^+^, and direct oxidation (Gadd. 2010; Wang YY. et al., 2020). Secondly, the organophosphorus source is solubilized by the production of extracellular enzymes, such as phytase, phosphatase/carbon phosphorus lyase (Rawat et al., 2020). It is noteworthy that PSM is widely used for solidification and stabilization of HMs in soil because the PO_4_^3-^ released by PSM can combine with HMs to form stable phosphate precipitation. In addition, PSM can also repair HMs pollution through adsorption and transformation. For instance, PSM is capable of biosorption, accumulation and complexation of HMs ions by cells themselves (extracellular adsorption, surface adsorption and intracellular absorption), surface functional groups and secreted organic substances (extracellular polymers, glutathione, etc.) (Ahemad, 2015). Alternatively, the valence of HMs is changed and their toxicity is reduced by the metabolic action of the PSM themselves (e.g., redox, methylation, etc.) (Gadd, 2004; Vassileva et al., 2010). However, unstable HMs forms (such as biosorption, complexation, etc.) that remain in the soil for a long time will inevitably be subject to environmental changes and transform again into bioavailable forms. Therefore, mineralization precipitation is the key to guarantee that PSM reduce the mobility and bioavailability of HMs in the soil, which gives PSM a place in the field of microbial solidification and stabilization remediation.

## 2 The solubilizing effect of PSM makes it difficult to remediate high concentrations of soil HMs pollution alone

Although PSM have a good remediation effect on soil HMs, the environment with high concentrations of HMs still seriously threatens the normal microbial metabolism process (Chen X. et al., 2020). For example, HMs ions can damage protein folding or the combination of cofactors and enzymes by binding with sulfhydryl groups on proteins, thus damaging the normal biological activity of proteins (Sharma and Melkania, 2018). Also, the hydroxyl radicals and reactive oxygen species (ROS) formed by the transition metals participating in Fenton reaction can also destroy biological macromolecules (He et al., 2017). Most notably, the solubilization of PSM is a “double-edged sword”. Organic acids secreted by PSM, such as formic acid, acetic acid, citric acid, oxalic acid, etc., can cause changes in soil pH and redox potential, and promote the weathering and dissolution of HMs minerals in soil (Ahemad, 2015). The solubilizing effect of PSM on HMs undoubtedly greatly increased the difficulty of PSM to repair HMs in soil alone. Herein, seeking a facile but efficient technology to make a synergistic interaction with PSM for boosting of HMs remediation efficiency is challenging but of great significance.

## 3 Biochar is a green and sustainable material that can assist phosphorus-dissolving microorganisms to remediate HMs in soil

### 3.1 Biochar is a promoter to enhance soil microbial remediation

Biochar is a carbon-rich material obtained by pyrolysis of biomass under anoxic conditions, which exhibits excellent adsorption and solidification effects on soil HMs. Multiple studies have demonstrated that surface complexation, electrostatic adsorption, ion exchange, mineral precipitation, etc. are the main mechanisms for the adsorption of HMs by biochar (Chen et al., 2022). As an excellent green sustainable material, biochar still has long-term stability to HMs, even when it faces the effects of aging in the environment (Wang L. et al., 2020), which is certainly a guarantee for its own application. On the other hand, adding biochar to soil will have a positive impact on soil environment, including soil aggregate, pH value, nutrient conservation, and improvement of microbial community (Shaaban et al., 2018). It is of interest that the porous structure of biochar can provide a good refuge for microorganisms inside the soil from HMs attacks. Meanwhile, the rich nutrients of biochar itself can also guarantee the reproduction of microorganisms (Cooper et al., 2020). Blanco-Vargas et al. (2022) also confirmed that biochar is an excellent biological inoculant to support the growth of phosphate-solubilizing bacteria. Therefore, the use of biochar to improve the efficiency of soil microbial remediation is a more effective strategy.

### 3.2 Toxicity attenuation and mineral precipitation mechanisms of biochar are key to the protection of phosphorus-dissolving microorganisms

**FIGURE 1:**
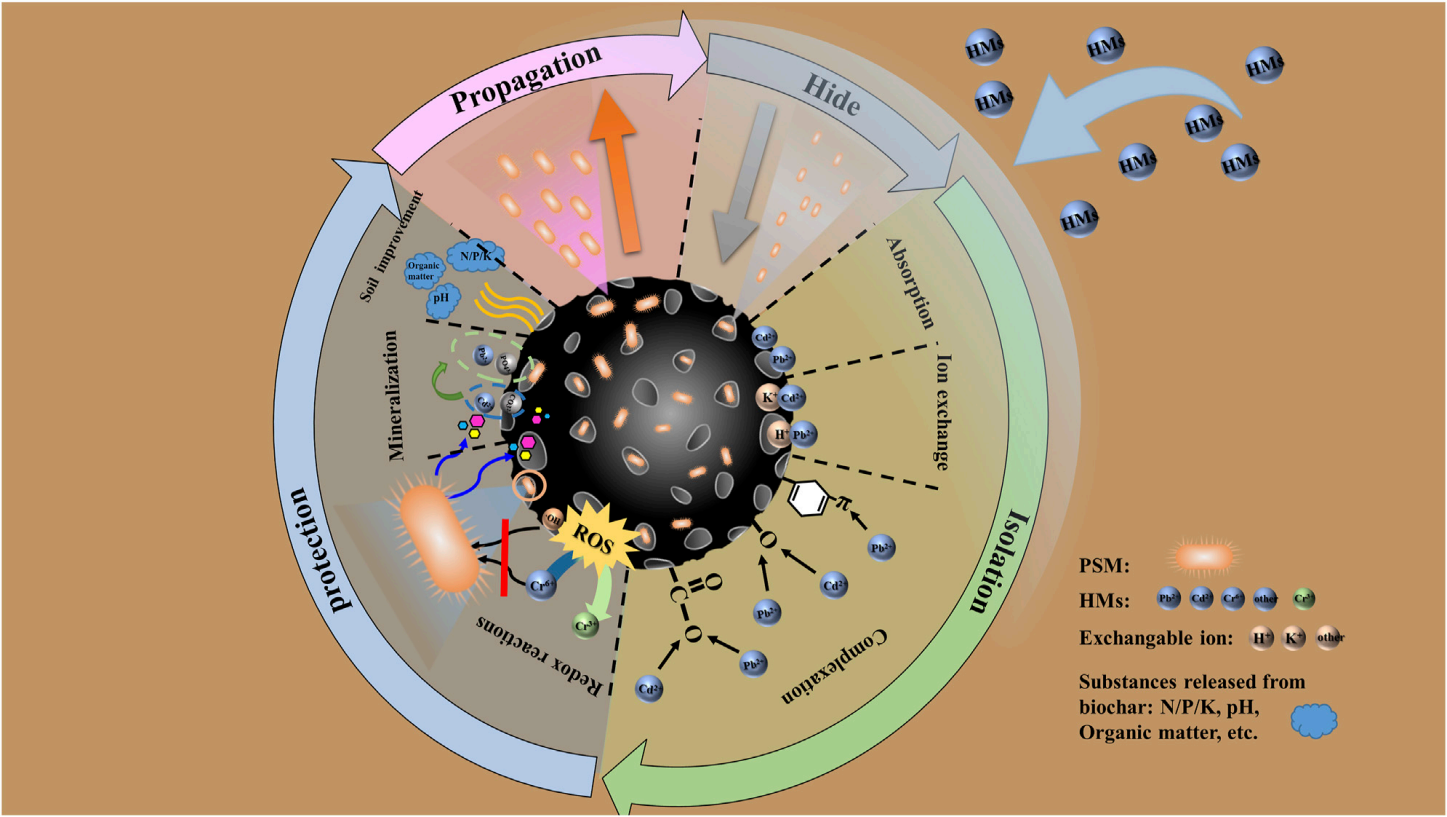
Schematic diagram of the mechanism of biochar-assisted phosphorus solubilizing microorganisms for remediation of heavy metals contamination in soil.

As mentioned earlier, it is because of its ability to reduce the transport, leaching and biotic stress of potentially toxic elements in soils that biochar has been successfully applied to assist microbial remediation of HMs contamination in soil. For example, pine cone biochar enhanced inorganic ion transport and metabolic rate by increasing the number of bacilli and immobile bacteria, thus in promoting the passivation and stabilization of HMs (Lan et al., 2021). Sludge and rice husk biochar improved phosphorus solubilizing bacteria (*Enterobacter* sp.) remediation efficiency and resistance to Pb^2+^ (217%–700%) and Cd^2+^ (85%–201%) under high concentration (1,000 mg/L) stress (Chen et al., 2019; Chen et al., 2020 H.). The combination of waste fungus chaff-based biochar and *Herbaspirillum huttiense* was able to solidify 85.5% Cu^2+^ and 64.4% Zn^2+^ (total Cu^2+^ and Zn^2+^ in background soil: 218.2 mg/kg and 427.1 mg/kg) (Zhang X. et al., 2022). *Bacillus cereus* with biochar converted 94.22% of Cr(VI) to Cr(III) in the soil and increased the residual Cr by 60% (Chen et al., 2021). Arbuscular mycorrhizal fungi and biochar enhanced the immobilization of Cr and reduced the Cr(VI) concentration by 0.3%–64.5% (Chen et al., 2022). Some scholars have also used Fe modified biochar with PSM to mineralize Pb^2+^ to Pb_5_(PO_4_)_3_Cl and Pb_5_(PO_4_)_3_OH (Teng et al., 2019). In general, the protection mechanism of biochar for PSM can be divided into four aspects: hiding, isolation, protection, and proliferation Figure 1.

First, biochar has abundant grooves and large pores on the surface and inside respectively, which allow microorganisms to hide in them (Zhang R-H. et al., 2022). Secondly, biochar reduces the biological effectiveness of HMs through surface adsorption and complexation control mechanisms (e.g., cation exchange, functional groups and C-π interactions, etc.), which sufficiently reduce the exposure of HMs to microorganisms (Chen et al., 2022; Gong et al., 2022). Thirdly, the redox effect of biochar itself can change the valence state of variable HMs (Cr^6+^, As^3+^, Hg^2+^) and accelerate electron transfer, which weaken the toxicity of toxic HMs (Gong et al., 2022; Lin et al., 2023). Also, biochar is able to induce the toxic groups (ROS) caused by the valence shift of HMs in advance outside the cell. Most importantly, biochar completely prevents HMs stress on PSM by providing C, P and other elements to form stable mineral precipitates with HMs (including those dissolved by PSM) (Chen et al., 2019; Lai et al., 2022). In addition, biochar can improve the physicochemical properties of the soil such as organic carbon, pH, CEC, agglomerates, etc., which may lead HMs to the formation of organic complexes to be immobilized (Zheng et al., 2022; Fu et al., 2023). For example, biochar can improve nutrient retention and reduce leaching by increasing soil CEC (Laird et al., 2010), thereby providing nutrient reserves for microorganisms. Biochar provides lasting benefits to microorganisms by slowly releasing its own dissolved organic carbon (a nutrient for microorganisms) (Zheng et al., 2022). Finally, the protected PSM multiply in large numbers by virtue of nutrients from biochar. This allows the bacteria to dissolve the inorganic phosphate source (e.g., apatite minerals) or the organic phosphate source (e.g., phenolphthalein diphosphate, phytate, glycerophosphate) again to mineralize the heavy metals, thus sustaining the bioremediation effect (Jiang et al., 2020). On the other hand, biochar is used by PSM as a platform to form a fortress for solidifying and stabilizing HMs. Considering the dissolution/release of HMs by bacteria themselves, the mechanism of toxicity attenuation and stable mineral precipitation provided by biochar enables PSM to cope with toxic stresses of both variable (Cr, Hg, Cu, As, etc.) and non-variable (Cd, Zn, etc.) HMs. In particular, biochar weakening the toxicity mechanism of variable heavy metals can give microorganisms the time to resist the stress. For example, biochar directly reduces Cr(VI) to the less toxic Cr(III) *via* phenolic-OH (Xu et al., 2019). In conclusion, PSM have the potential to remediate high concentrations of HMs contamination with the support of biochar, and biochar with high P content (sludge, pig manure, etc.) can give more support.

## 4 Prospects

PSM have gradually attracted attention as an important tool for soil remediation of HMs contamination. Especially in recent years, relevant studies have confirmed that PSM can be applied in the remediation of agricultural soil and industrial sites with many different HMs. Although a number of studies have confirmed its mechanism of remediation of HMs, most of them have found that the application of PSM is often considerably less effective in scenarios with high concentrations of pollution. Biochar, as a green and friendly soil remediation agent, has been shown to enhance the survival of PSM in high concentrations of HMs. However, most biochar co-remediation with PSM has been performed in the laboratory and for a single HMs contamination. Therefore, future PSM combined biochar remediation should be tested in actual or simulated application scenarios, and used for remediation of multiple HMs combinations of pollution. In addition, the incorporation of various environmentally friendly modification techniques to assist biochar in promoting the remediation function of PSM is also a promising approach. On the other hand, the affinity of biochar with microorganisms and effectiveness of their practical applications will also be a challenge for its joint application. There is a need for us to develop large-scale applications of combined remediation technologies and to enhance the scenarios in which biochar combined with PSM remediation technologies can be applied. Finally, the riskiness of the remediated soil for HMs at long time scales also needs to be considered in order to minimize adverse consequences.
